# First person – Christine Lehner

**DOI:** 10.1242/dmm.043547

**Published:** 2019-12-16

## Abstract

First Person is a series of interviews with the first authors of a selection of papers published in Disease Models & Mechanisms, helping early-career researchers promote themselves alongside their papers. Christine Lehner is first author on ‘Tenophages: a novel macrophage-like tendon cell population expressing CX3CL1 and CX3CR1’, published in DMM. Christine is a researcher in the lab of Andreas Traweger at Paracelsus Medical University, Salzburg, Austria, investigating the mechanisms leading to tendinopathies.


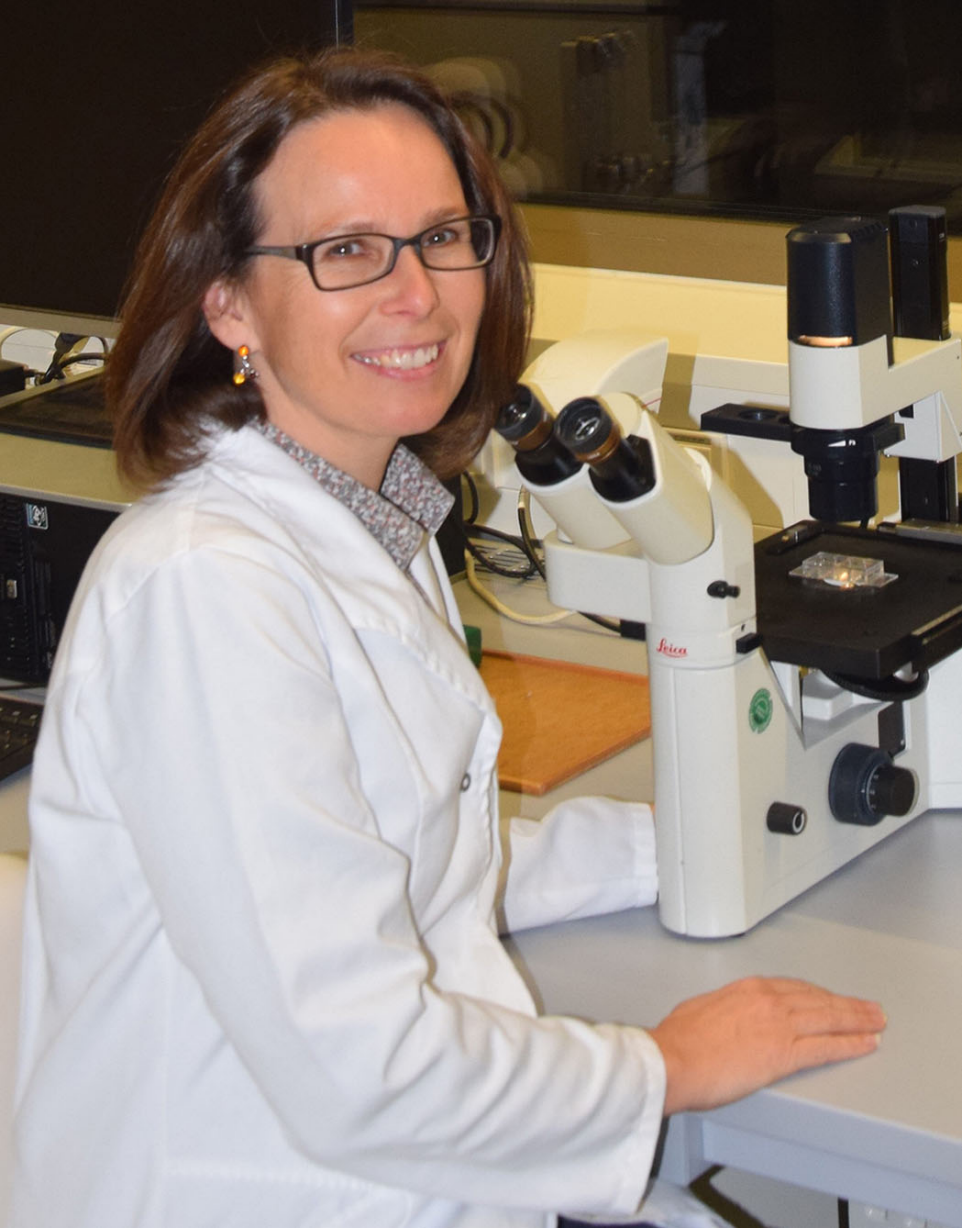


**Christine Lehner**

**How would you explain the main findings of your paper to non-scientific family and friends?**

The mechanisms leading to tendon degeneration are still poorly understood and therefore treatment options remain limited. Recently, there is increasing evidence that inflammatory cells are involved. So far it has been assumed that these cells come from outside the tendon tissue, e.g. from the bloodstream. In our work, we show that cells with immune cell character are already present in the tissue and that it is the tendon cells themselves that express these immune cell markers. In order to address the dual nature of these cells, we have therefore coined the term ‘tenophages’. In addition, we were able to show that these cells express specific proteins that were previously not associated with tendons at all. Interestingly, these proteins have been playing a role in the sprouting of blood vessels, cell division and scar formation in other tissues, all of which are hallmarks of tendinopathies. Therefore, this newly discovered cell population could prove to be a novel therapeutic target.

**What are the potential implications of these results for your field of research?**

With the newly described tenophages, which express a receptor-ligand axis well described in other tissues, we have introduced new players into the existing group of candidates known to be crucial for tendon homeostasis. The definition of their role and function in health and disease will be a challenge for future research, to open up promising new treatment options, as there are very specific drugs that are already clinically approved and suitable for therapeutic interventions.

**What are the main advantages and drawbacks of the model system you have used as it relates to the disease you are investigating?**

An advantage of working with rodent tissue is that we have healthy, intact tissue at hand, which, in our case, was key, since we aimed to determine the presence of immune cells in the healthy state. In contrast, to receive healthy human tissue is much more challenging. A drawback of the animal model we used is that there is still no satisfactory disease model for tendinopathy.

“Finding immune cells inside the dense tendon core was quite surprising, and even more so was that these cells turned out to be tendon cells, expressing both tendon-specific and immune cell-related markers.”

**What has surprised you the most while conducting your research?**

Before we started our study, we did not know whether immune cells were present in the tendon itself at all. We expected to find perivascular macrophages, immune cells located near blood vessels and known to be present in other tissues. Finding immune cells inside the dense tendon core was quite surprising, and even more so was that these cells turned out to be tendon cells, expressing both tendon-specific and immune cell-related markers.

**Describe what you think is the most significant challenge impacting your research at this time and how will this be addressed over the next 10 years?**

The characterization of tendon cells and distinguishing them from other ‘fibroblasts’ is certainly a major challenge. The limited number of markers expressed exclusively by tendon cells is still a limiting factor in tendon research. Another major challenge in our work is that we are still lacking an adequate animal model for tendinopathy. Most of the published models only mimic symptoms, but do not necessarily address disease etiology, which is still largely unknown, or are extremely elaborate and raise ethical issues. We hope that in the future there will be more mechanistic knowledge about the etiology of tendinopathies and that more suitable animal models for tendinopathy will be established.

**Figure:**
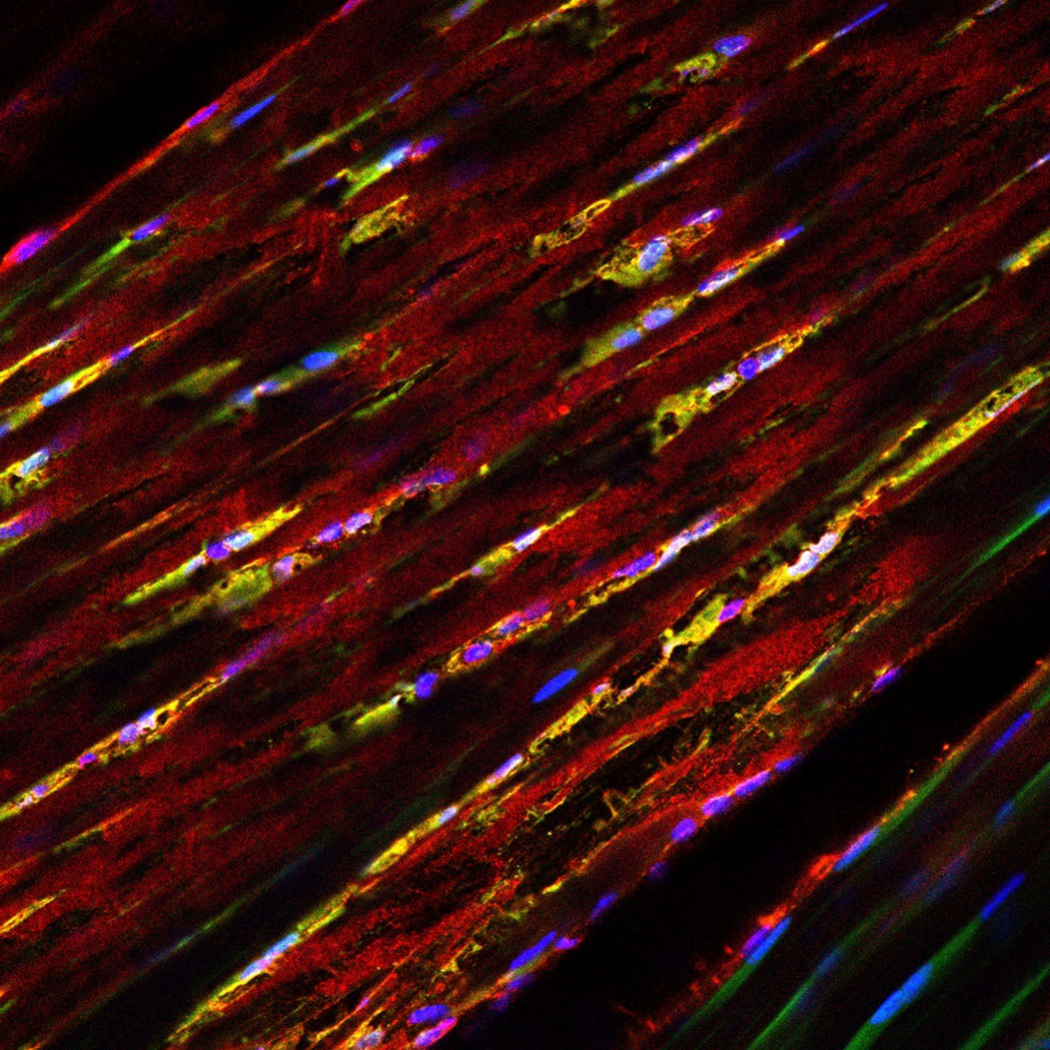
**Immunofluorescence image of a cryosection from an Achilles tendon of an *Scx-GFP* reporter mouse, showing the expression of GFP (green) and CX3CR1 (red).**

“[…] it is important that mentors actively introduce young scientists to the scientific community at conferences or meetings so that they become part of it and receive networking support.”

**What changes do you think could improve the professional lives of early-career scientists?**

In my opinion, the availability of funding opportunities, in which young scientists can apply for scholarships (also for smaller ones) to support their research projects, in combination with supervision by established scientists is decisive for improving the professional lives of young scientists. With support and advice from experienced mentors on project design and application, young scientists benefit from many years of experience, avoid beginners’ mistakes and increase the likelihood of project funding. This in turn encourages young scientists to pursue their research interests and makes them more self-confident and (at least financially) independent. In addition, it is important that mentors actively introduce young scientists to the scientific community at conferences or meetings so that they become part of it and receive networking support.

**What's next for you?**

As a next step, I am working for the postdoctoral qualification at our university. Regarding my research, I will certainly follow up the findings just published since every finding entails hundreds of new questions, and it is the in-born curiosity of every scientist to demand the answer to at least some of these questions.
